# Farmers’ perceptions and management of citrus fungal diseases in Benin

**DOI:** 10.1016/j.heliyon.2024.e32775

**Published:** 2024-06-13

**Authors:** Goudjo Habib Toessi, Rachidatou Sikirou, Elisée Georges Dadé Ler-N'ogn Amari, Esaïe Gandonou, Afio Zannou, Daouda Koné

**Affiliations:** a African Excellence Center on Climate Change, Biodiversity and Sustainable Agriculture (CEA-CCBAD), Félix Houphouët-Boigny University, 22 BP 582, Abidjan, Cote d'Ivoire; b Laboratory of Biotechnology, Agriculture and Valorization of Biological Resources, UFR Biosciences, Félix Houphouët-Boigny University, 22 BP 582, Abidjan, Cote d'Ivoire; c Laboratory of Crop Protection (LDC), National Institute of Agricultural Research of Benin (INRAB), 01 BP 884, Cotonou, Benin; d Laboratory of Agroeconomics and Agribusiness (LAGEC-B), University of Abomey-Calavi, 01 BP 526, Cotonou, Benin

**Keywords:** Citrus, Fungal diseases, Farmers' perceptions, Benin

## Abstract

**Background and aim:**

Citrus production represents an important activity for the national economy and a source of income for farmers in Benin. However, fungal diseases are a major constraint to production intensification. The aim of this study is to assess farmers’ perceptions on citrus fungal diseases in production areas in Benin.

**Methods:**

A survey was conducted among 417 farmers between July and December 2021 in four major citrus-producing agro-ecological zones (zones V, VI, VII and VIII) to collect their perceptions, knowledge and management practices of citrus fungal diseases.

**Results:**

Farmers reported that fungal diseases are one of the main constraints to citrus production, including black spot, anthracnose, brown rot, sooty mold and fruit rot. Among them, black spot disease is the most severe, causing damage to production. According to farmers, symptoms appear on fruit after fruit set, with a very remarkable presence and high incidence at maturity. Although farmers are most of times aware of the damage caused by fungal diseases with adverse consequences on their income, they have a poor knowledge of appropriate phytosanitary products to manage these diseases. Indeed, the majority of farmers (>60 %) use chemical insecticides, which they reported to be ineffective against citrus fungal diseases. Although chemical insecticides are their only recourse, almost 40 % use nothing to control these diseases. Farmers stated that climatic variability is a factor favoring the development of diseases, leading to reduced production.

**Conclusions:**

Among the several citrus fungal diseases, black spot is perceived as the most damaging, causing greater yield losses under favorable conditions, coupled with an almost total absence of appropriate control methods. This study contributes to the reorganization of the citrus industry and to decision-making on capacity building for farmers in terms of orchard pest protection, in order to guarantee better production of marketable and exportable fruit.

## 1 Introduction

Citrus fruits originate from Southeast Asia and are an economically important fruit crop worldwide [1]. They are currently grown in over 140 countries, and the main commercial species are oranges (*Citrus sinensis* L. Osbeck), mandarins (*Citrus reticulata*), lemons (*Citrus limon*), limes (*Citrus aurantiifolia*) and grapefruits (*Citrus x paradisi*), belonging to the Citrus genus of the Rutaceae family. Subtropical regions are the main areas of commercial production, where the best quality citrus fruits are grown [1,2]. Global citrus production is estimated at over 158 million tons in 2020 [3]. In tropical Africa, citrus is grown largely by small-scale farmers for local consumption and export [4]. On this continent, an annual production of over 20 million tons was harvested in 2018 from an area of more than 3.3 million hectares of citrus [3]. From a nutritional point of view, citrus fruits are rich in essential nutrients and energy, which are beneficial to human health [5]. They contain a variety of secondary metabolites and bioactive compounds, such as coumarins, alkaloids, carotenoids, limonoids, phenolic acids, flavonoids and essential oils, which have functional health benefits [[6], [7], [8]]. Citrus fruits are also rich in vitamins C, A and E, minerals, pectins and other beneficial phytochemicals, making them an important basis for a balanced diet [9].

Among the various sectors, the citrus industry has been considered by political leaders for several years as an option for achieving food and nutritional security in Benin. Citrus production represents an important activity in the national economy and is a source of income for farmers [10]. In Benin nearly 15500 tons of citrus fruit were produced with an area of 5542 ha in 2020 [3]. Despite favorable climatic conditions, farmers are faced with a number of phytosanitary problems, including diseases and insect pests. Diseases, especially fungal ones (black spot, anthracnose, fruit rot, etc.), are currently making their appearance in citrus orchards and are a major constraint on the development of the citrus industry in Benin. Their damage to fruit affects yield and consequently farmers’ income with low value on local and international markets [11,12]. Citrus black spot disease caused by *Phyllosticta*, is now a serious threat to citrus production worldwide [13]. In Ghana, brentu et al. [13] reported that this disease caused around 80 % of yield losses. In South Africa, the majority of attacked fruit from trees not protected against black spot disease were declared unfit for export, causing over 80 % of economic loss [14]. Furthermore, the control of these pests in general and fungal diseases in particular is essentially based on the use of chemical products. In addition to being very costly for small citrus farmers and detrimental to health and income, these chemicals contribute significantly to greenhouse gas (GHG) emissions. Excessive pesticide use increases the production of nitrous oxide in soils. Many pesticides produce ground-level ozone, a greenhouse gas harmful to humans and plants [15].

In addition, the influence of climate variability is a very important aspect in pest management. Indeed, climatic variability, reflected by changes in average temperatures, reduced annual rainfall, irregular rainfall distribution and prolonged periods of drought, can affect the productive quality of plants and potentially cause their death [16]. It creates favorable conditions for the emergence and aggressiveness of disease-causing pathogens, thus increasing their level of damage. Deberdt et al. [17] reported that climatic factors can change the nature of microorganisms, transforming them into opportunistic pathogens. Previous work on pathogenic microorganisms has shown that plants weakened or stressed by environmental factors, become more vulnerable to microorganisms [18,19]. In order to be informed to help policies in decision-making, it is important to document farmers' knowledge of the pests affecting citrus in the different agro-ecological zones (AEZ) of citrus production, the extent of their damage on production and their incomes. The aim of this study is to assess farmers’ perceptions and practices in the management of citrus-infected fungal diseases in production areas in Benin.

## 2 Materials and methods

### 2.1 Study areas

**Fig. 1:**
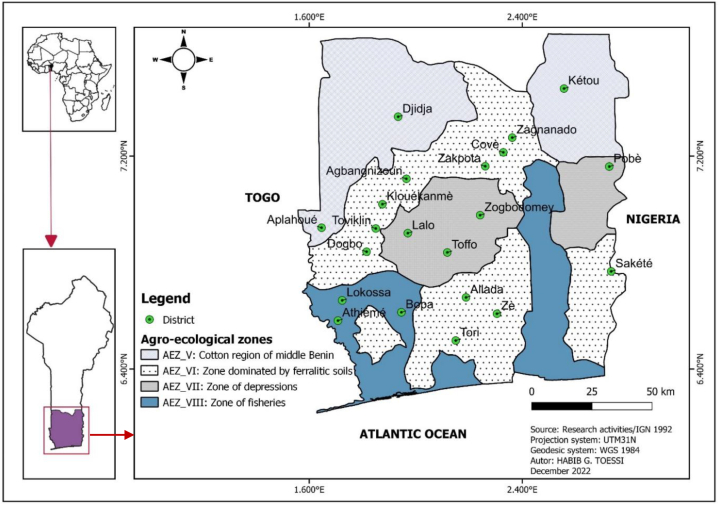
Geographical location of study areas.

The present study was carried out in the citrus-growing area of Benin. It covered four agro-ecological zones (AEZs), namely the cotton-growing zone of central Benin (AEZ V), the barre land zone (AEZ VI), the depression zone (AEZ VII) and the fishing zone (AEZ VIII) (Fig. 1). Predilection of citrus production, agro-climatic characteristics and accessibility were the main criteria for study site selection (Table 1).

**Table 1:** Characteristics of agro-ecological zones.

AEZ	Definition	Climate	areas (km^2^)	rainfall (mm)	Annual temperature (°C) (SD)
**V**	Central Benin cotton zone	Sudano-Guinean climate	31722	1100	14–29
**VI**	Bar land zone	Sudano-Guinean or sub-equatorial climate	6373	800–1400	25,3–29,7
**VII**	Depression zone	Sub-equatorial climate	2564	800–1300	27–31
**VIII**	Fishing zone	Sub-equatorial climate	3151	1000–1400	25–30

SD= Standard Deviation.

### 2.2 Sample size

An exploratory study was carried out in the study areas prior the real survey, in order to determine the sample size to be considered, using a normal approximation to the binomial distribution proposed by Dagnelie [20].

$$N=\frac{\left[\left(U_{1-\alpha /2}\right)²\times P\;\left(1-p\right)\right]}{d^{2}}$$

**U =** value of the normal random variable for the probability value of **1-α/2**;

**P =** probability of **1-α/2**, **α =** risk of error, d = margin of estimation error.

For **α =** 5 %, probability **p =** 1-α/2 = 0.975 and **U**_**1**_**-α/2 =** 1.96.

**p** is the proportion of people engaged in citrus production in the study area and **d** the margin of estimation error, retained at 8 % in this study [21]. Based on the p-values derived from the results of the exploratory phase of the study, 417 farmers were surveyed in the four AEZs: 55 for AEZ V, 236 for AEZ VI, 72 for AEZ VII and 54 for AEZ VIII. Importance of citrus area sown was the selection criteria for farmers.

### 2.3 Data collection

The study was conducted from June 2021 to December 2021. As the majority of farmers are illiterate, appropriate approaches were developed to ensure better understanding and effective participation. Data were collected using a semi-structured questionnaire that was directly and orally administered to individual citrus farmers in the four AEZs. The interviewers asked the farmers questions in local languages and recorded their responses. Information collected covered farmer's demography and socio-economic characteristic (age, education level, gender, marital status, ethnic group, mode of access to land and membership of an association, other activities, family size), citrus orchards characteristics (area, density and age of orchards, citrus species), expression of fungal diseases, periods and stages of fungal disease appearance, for how long (year) they have recorded the presence of fungal diseases, disease management methods, farmers' perception of climatic variability (temperature, relative humidity and rainfall). Farmers were also asked to determine, through the questionnaire, at what time (month) of the year they generally notice an attack of fungal diseases affecting more than 50 %, less than 50 %, or no attack at all on their citrus plants. To avoid confusion between the symptoms of fungal diseases and insect pest, photos of symptoms of different citrus fungal diseases were attached to the questionnaire and presented to the farmers. This allowed farmers to indicate their responses by pointing to the corresponding photos.

### 2.4 Statistical analysis

Data were analyzed using R software version 4.0.2 [22]. The socio-economic characteristics of the farmers were summarized using cross-tabulations. The Chi-square test was used to test the association between socio-economic characteristics and agro-ecological zones. Descriptive statistics (mean ± standard error) were used to summarize orchard age, area and density by AEZ and variety. The general linear model (GLM) and analysis of variance (ANOVA) followed by Tukey's post hoc test, as performed in the ‘multcomp’ package [23], were used to test the difference in age and area between zones and between species. The Kruskal-Wallis test was used to determine disease occurrence year numbers. Bar charts and heat maps were used to highlight the periods and stages of appearance of each disease inventoried by zone and specie. Cross-tabulations, proportions and graphs were produced to summarize farmers' perceptions of the effect of climatic variability on citrus production and disease development.

## 3 Results

### 3.1 Characteristics of citrus farmers in Benin

Data analysis showed that 17.7 % of farmers were less than 40 years old and 82.3 % were over 40. The distribution of farmers by gender revealed that men, with a proportion of 92.8 %, were predominant in the citrus sector. 42.8 % of farmers were illiterate, 34 % with primary education, 21.1 % with secondary education and only 2.2 % with higher studies. The "purchase" mode of land acquisition was the most dominant (63.9 % of farmers), followed by "inheritance" (30.6 %). In terms of matrimonial status, over 99 % of farmers were married. The majority of farmers surveyed (85.4 %) belonged to no agricultural association (Table 2).

**Table 2:** Socio-economic characteristics of farmers.

Characteristics		Agroecological zone				Overall	Percentage (%)	p-value
		V	VI	VII	VIII			
Gender	Male	47	237	63	41	388	92,8	0.066
	Female	1	25	1	3	30	7,2	
Age	<40 years	8	39	19	8	74	17,7	0.089
	40–50 years	21	95	19	20	155	37,1	
	>50 years	19	128	26	16	189	45,2	
Marital status	Married	47	261	64	44	416	99,5	0.415
	Widowed	1	1	0	0	2	0,5	
Ethnic group	Adja	21	49	24	9	103	24,6	**< 0.001**
	Fon	17	158	17	2	194	46,4	
	Sahouè	0	0	0	19	19	4,5	
	Yoruba	0	1	0	0	1	0,2	
	Tori	0	15	0	0	15	3,6	
	Kotafon	0	3	17	14	34	8,1	
	Others	10	36	6	0	52	12,4	
Position in the household	Head of household	47	235	62	41	385	92,1	0.573
	Spouse	1	20	1	3	25	6,0	
	Son/Daughter	0	6	1	0	7	1,7	
	Related	0	1	0	0	1	0,2	
Educational level	None	23	118	30	8	179	42,8	**0.003**
	Primary school	13	90	20	19	142	34,0	
	Secondary school	11	53	11	13	88	21,1	
	University	1	1	3	4	9	2,2	
Land access mode	Purchase	24	167	39	37	267	63,9	0.141
	Donation	2	13	3	1	19	4,5	
	Legacy	22	78	22	6	128	30,6	
	Renting	0	3	0	0	3	0,7	
	Other	0	1	0	0	1	0,2	
Member of an association	No	42	218	49	42	351	84,0	**0.048**
	Yes	6	44	15	2	67	16,0	
Member of an association of citrus producers	No	47	205	61	44	357	85,4	**< 0.001**
	Yes	1	57	3	0	61	14,6	

### 3.2 Distribution of orchards by age class and area according to agro-ecological zone

**Fig. 2:**
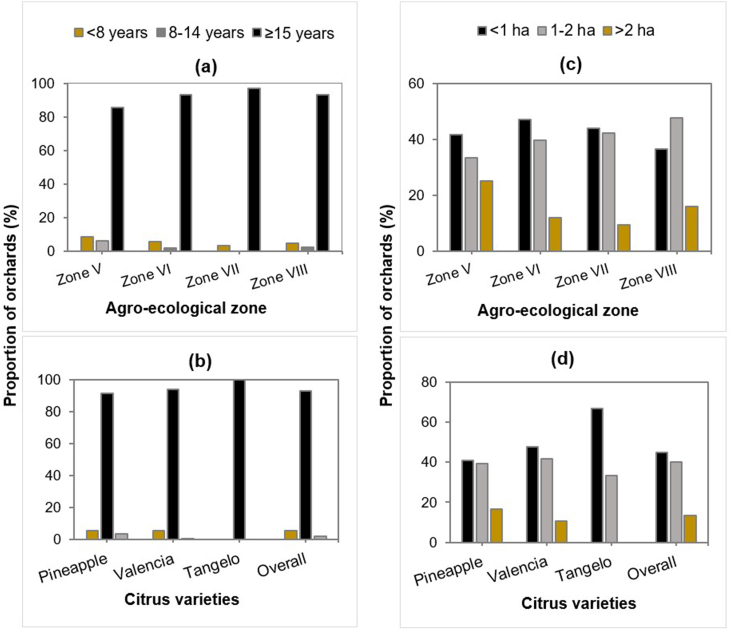
Distribution by orchard age (**a**, **b**) and area (**c, d**) across agro-ecological zones and citrus species.

Irrespective of agro-ecological zone or citrus species, the age of plantations ranged from 4 to 30 years, with an average of 10.33 ± 0.22 years. The distribution of orchards by age class revealed that over 85 % of orchards were over 15 years old (Fig. 2a and b). Overall, more than 50 % of farmers have an area of less than 1 ha (Fig. 2c and d). Furthermore, in all the AEZs inspected, orchards exceeding 2 ha were only Pineapple and Valencia varieties, while over 70 % of tangelo orchards did not exceed 1 ha. Analysis of the data relative to citrus orchard age and area showed that there was a significant difference between orchard age (p < 0.001) and area (p = 0.05) depending on the AEZs (Table 3).

**Table 3:** Orchard characteristics (age and size) by agro-ecological zone and citrus species.

	Orchard age (year)			Area of orchard (ha)		
	Range	mean ± SE	*p*	Range	mean ± SE	*p*
**Agroecological zones**						
Zone V	5–15	9.71 ± 0.42^b^	**<0.001**	0.12–10.00	2.25 ± 0.42^a^	**0.05**
Zone VI	4–30	11.24 ± 0.30^a^		0.03–19.00	1.42 ± 0.13^b^	
Zone VII	5–20	9.23 ± 0.41^b^		0.20–5.00	1.14 ± 0.13^b^	
Zone VIII	4–15	7.18 ± 0.39^c^		0.28–8.00	1.47 ± 0.24 ^ab^	
Overall	4–30	10.33 ± 0.22		0.03–19.00	1.47 ± 0.10	
**Citrus varieties**						
Pineapple	4–25	10.62 ± 0.30^a^	**0.342**	0.06–14.00	1.63 ± 0.15^a^	**0.11**
Valencia	4–30	10.02 ± 0.34^a^		0.03–19.00	1.33 ± 0.14^a^	
Tangelo	6–16	10.67 ± 0.93^a^		0.06–2.00	0.76 ± 0.27^a^	
Overall	4–30	10.33 ± 0.22		0.03–19.00	1.47 ± 0.10	

p = p-value, SE = standard error.

Means with the same letters in subscript are not statistically different at 5 % probability level.

### 3.3 Citrus species grown per agro-ecological zone

**Fig. 3:**
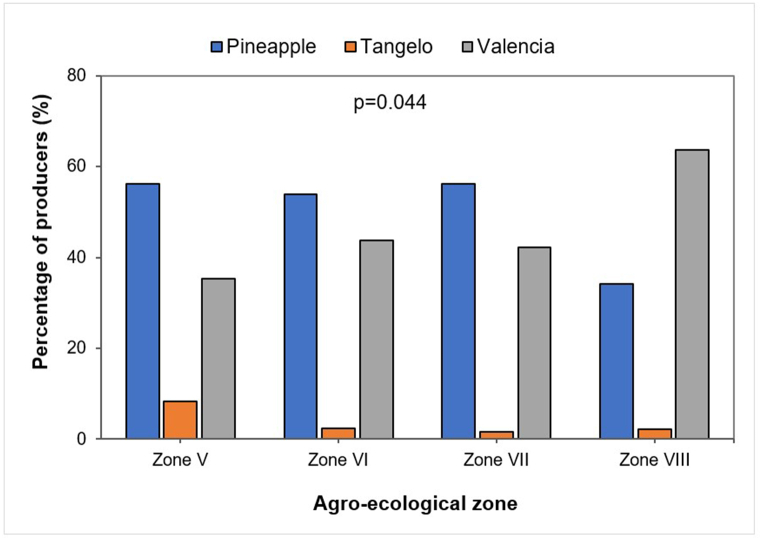
Distribution of citrus species across agro-ecological zones.

All the citrus orchards of the farmers surveyed were dominated by the Pineapple and Valencia orange varieties. Pineapple was the most widely grown variety in all the AEZs except AEZ VIII, where Valencia was grown in over 50 % of the orchards (Fig. 3). Tangelo was also produced in all agro-ecological zones, but in very small proportions. Fisher's exact test revealed a significant association between varieties grown and AEZs (p = 0.04).

### 3.4 Determination of orchard density per agro-ecological zone

**Fig. 4:**
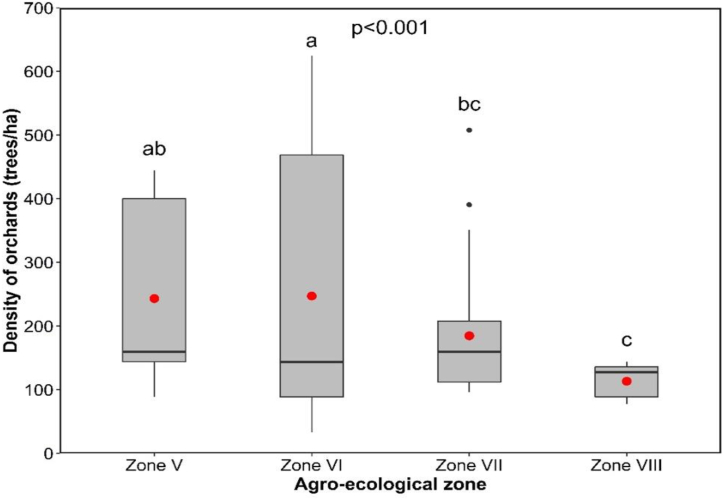
Citrus orchard density by agro-ecological zone.

Average orchard density was around 203 plants/ha, and varied significantly not only between AEZs but also within each AEZ (p < 0.001) (Fig. 4). AEZs V and VI had heterogeneous densities, while densities were more or less homogeneous in AEZs VII and VIII. Density was high, at around 247.32 plants/ha in AEZ VI, and lower, at around 113.37 plants/ha in AEZ VIII.

### 3.5 Farmers’ perceptions of orchard phytosanitary problems and pest management

**Fig. 5:**
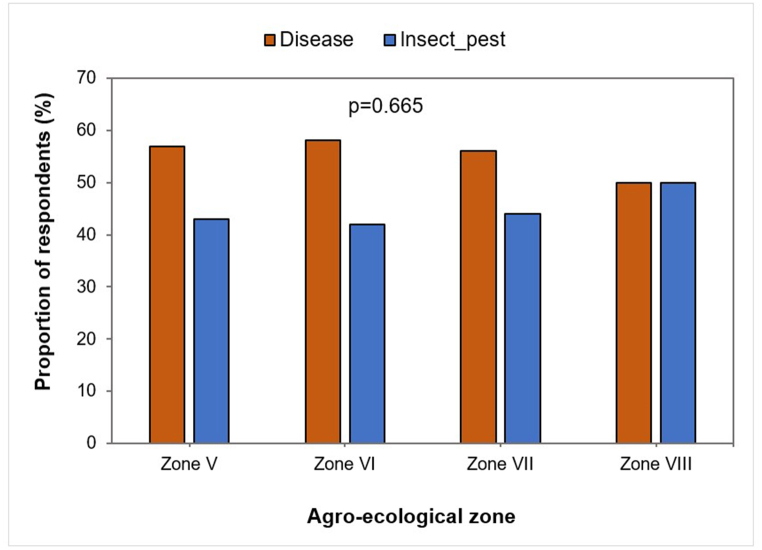
Proportion of respondents reporting biotic constraints linked to citrus production by agro-ecological zone.

For 50–58.1 % of farmers in most AEZs, diseases were a major constraint to citrus production. As for insect pests, 42–50 % of farmers were aware that they were also constraints to citrus production (Fig. 5). The occurrence of diseases or insect pests did not depend on the AEZ (p = 0.665).

### 3.6 Farmers’ perception of citrus fungal diseases

**Fig. 6:**
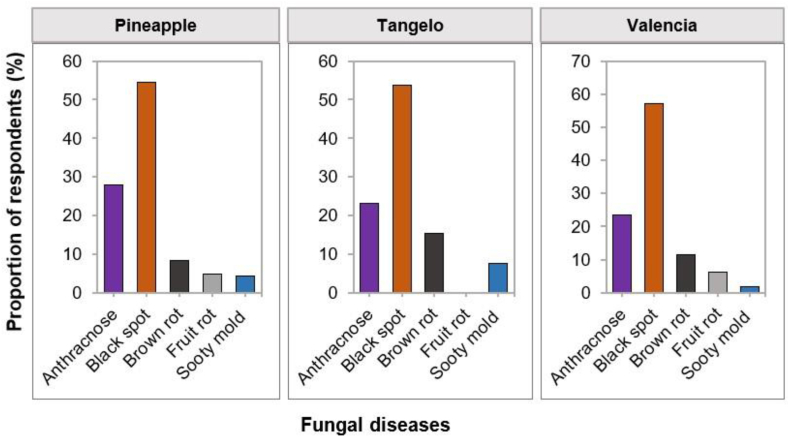
Proportion of farmers having observed the different fungal diseases in Pineapple, Tangelo, and Valencia orchards.

Referring to the fungal disease identification guides they have been given, the farmers identified fourcitrus fungal diseases mainly black spot, anthracnose, brown rot, sooty mold and fruit rot (Fig. 6). Of these diseases, more than half the farmers interviewed admitted that only black spot disease was most frequently observed in the orchards, and this was the case for all species. The number of years of disease appearance varied significantly from one disease to another (p = 0.05), and ranged from 1 to 20 years (Table 4).

**Table 4:** Observation year for citrus fungal diseases.

Diseases	Mean ± S.E	observation period (year)
Black spot	5.62 ± 0.22^a^	1–20
Anthracnose	5.50 ± 0.20^a^	1–18
Curvulariose	6.26 ± 0.37^b^	1–20
Fruit rot	6.41 ± 0.44^b^	2–20
Fumagin	5.25 ± 0.90^a^	1–20

SE = standard error.

Means with the same letters in subscript are not statistically different at 5 % probability level.

**Fig. 7:**
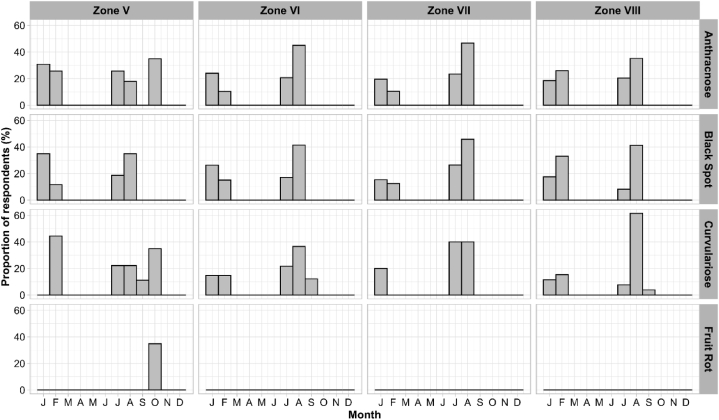
Periods when the farmers recorded the presence of fungal diseases according to agro-ecological zones.

**Fig. 8:**
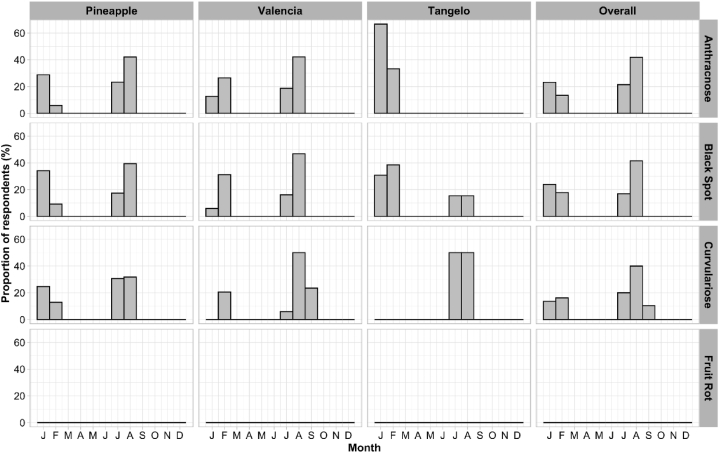
Periods when the farmers recorded the presence of fungal diseases according to citrus species.

**Fig. 9:**
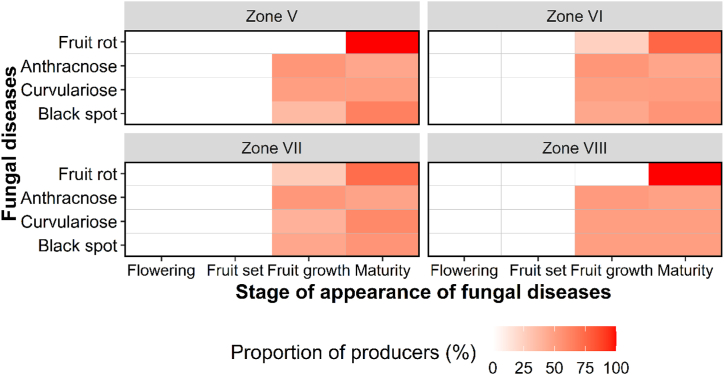
Appearance stages of fungal diseases across agro-ecological zones.

**Fig. 10:**
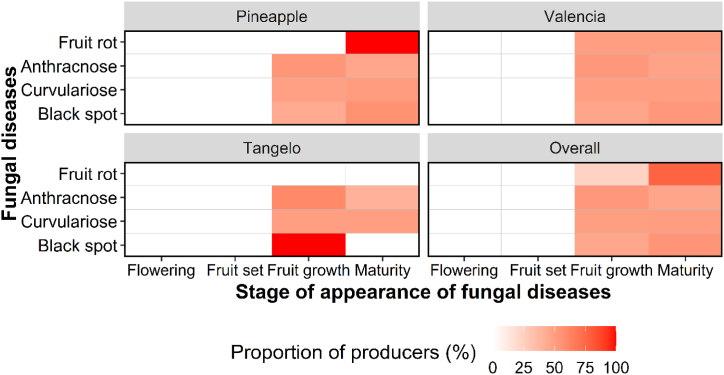
Appearance stages of fungal diseases according to citrus species.

**Fig. 11:**
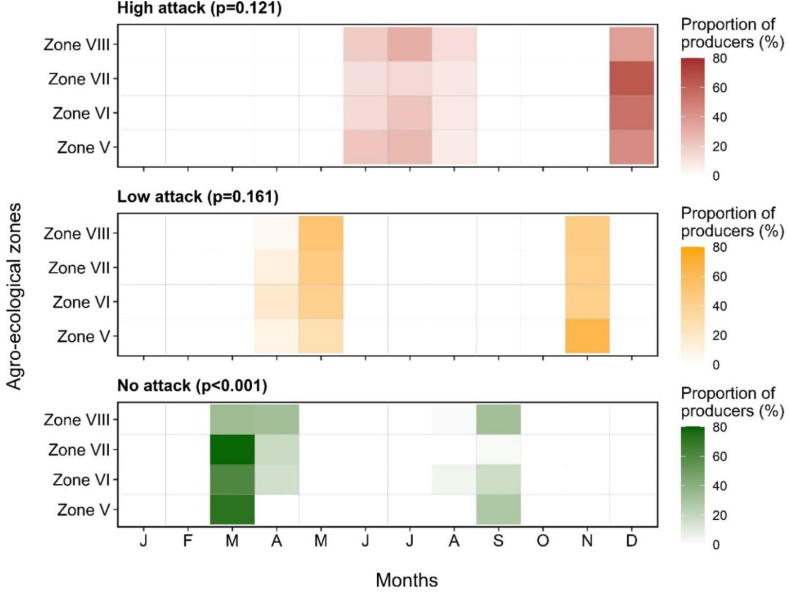
Different attack levels *per* period during the year according to farmers in agro-ecological zones.

The period of fungal disease appearance varied throughout the year, in January, February, July and/or August (Fig. 7, Fig. 8). Analyses of farmers’ perceptions revealed that, irrespective of zone and species, most diseases appeared from the fruit set stage through to ripening (Fig. 9, Fig. 10). Periods of high and low attack did not depend on AEZ (p > 0.05), unlike periods without attack (p < 0.001). High attacks of citrus fungal diseases were observed in June, July, August and December, while low attacks were observed in May and November. During March, April and September, farmers did not encounter any attacks (Fig. 11).

### 3.7 Farmers’ perceptions of fungal disease control methods

**Fig. 12:**
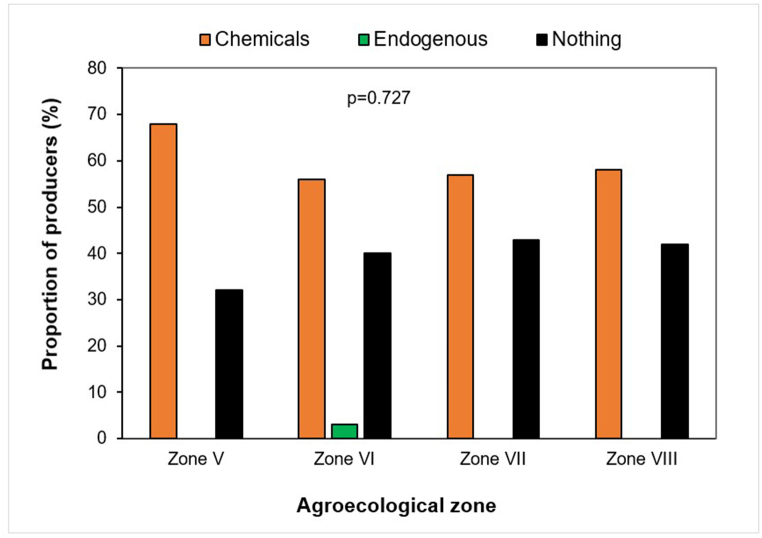
Proportion of farmers using methods (chemicals, endogenous, and nothing) to control fungal diseases by agro-ecological zone.

To control the diseases reported by farmers, 60 % of those interviewed had adopted chemical control. This control method did not vary from one AEZ to another (p = 0.727). Although these methods were their only recourse, almost 40 % of them used no method to control the diseases (Fig. 12).

**Fig. 13:**
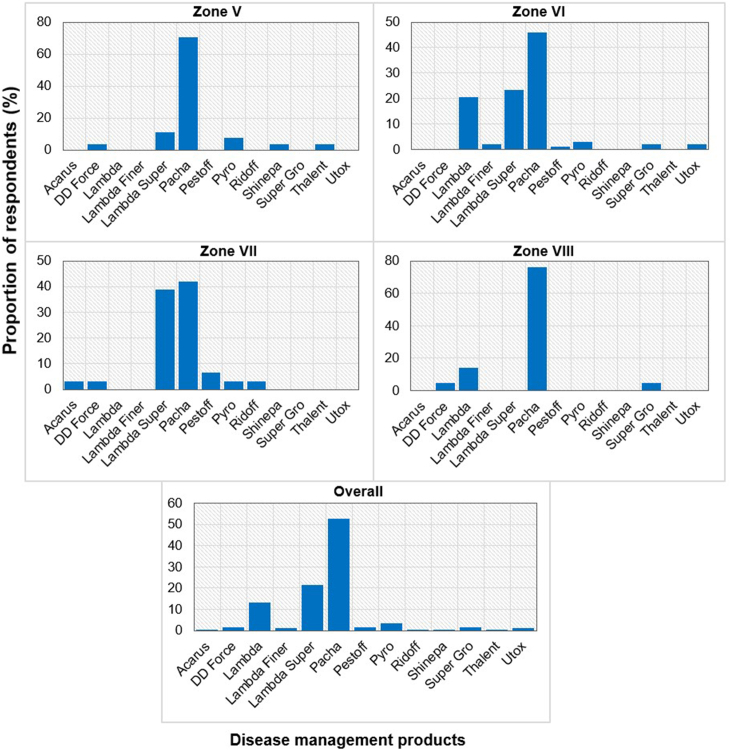
Distribution of chemical products frequently used to control citrus fungal diseases according to farmers in the 4 agro-ecological zones (Zone I, II, III, IV, and V) and overall.

In the management of citrus fungal diseases, farmers used various phytosanitary products, including Acarius 018 EC (Abamectin 18 g/L), DD Force (Dichlorvos 1000 g/L EC), Lambda Super 2.5 EC (Lambda-Cyhalothrin 25 g/L), Pacha 25 EC (Lambda-Cyhalothrin 15 g/L + Acetamiprid 10 g/L), Pyro FTE 672 EC (Cypermethrin 72 g/L + Chlorpyrifos-ethyl 600 g/L), etc. (Fig. 13). Among these products, 41.93–76.19 % of farmers reported "Pacha" as the chemical most used to control citrus fungal diseases in all AEZs. Pacha was followed by "Lambda Super", which was also well used in AEZ VII, by 38.71 % of farmers. According to the farmers, all the chemicals used were significantly ineffective against citrus fungal diseases (Table 5).

**Table 5:** Efficiency (%) of phytosanitary products used by farmers.

Phytosanitary products	High efficiency	Moderately efficient	Low efficiency	Ineffective
Pacha 25 EC (Lambda-Cyhalothrin 15 g/L + Acetamiprid 10 g/L)	0	0	17,92	82,08
Lambda Super 2,5 EC (Lambda-Cyhalothrin 25 g/L)	0	0	8,33	91,67
Pyro FTE 672 EC (Cyperméthrine 72 g/L + Chlorpyrifos-éthyl 600 g/L)	0	0	0	100
Pestoff	0	0	0	100
DD Force (Dichlorvos 1000 g/L EC)	0	0	0	100
Acarius 018 EC (Abamectine 18 g/L)	0	0	0	100

### 3.8 Farmers’ perception of the relationship between climate variability and fungal diseases

**Fig. 14:**
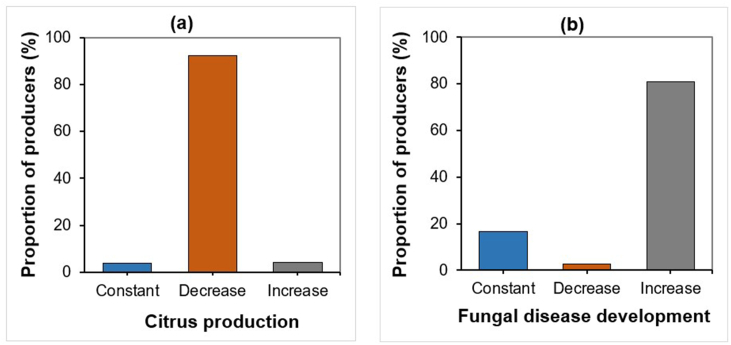
Climatic variability effect on citrus production (**a**) and fungal diseases development (**b**).

Indeed, the majority (92.2 %) of farmers reported a reduction in production due to rising temperatures and rare and variable rainfall periods (Fig. 14a). Similarly, 81 % of farmers indicated that this latter causes a significant increase in fungal disease outbreaks (Fig. 14b).

## 4 Discussion

Analysis of socio-economic characteristics indicates that the farmer community is dominated by older people. The great interest shown by older farmers can be explained by the fact that orchards represent, for them, a savings source for their retirement or period of inactivity. The average age of most orchards (around 86 %), regardless of agro-ecological zone or species, is greater than or equal to 15 years. This shows that Benin's citrus orchards are dominated by older plants, which will mostly enter the ageing phase in coming years. De Souza et al. [24] also reported that the average age of citrus orchards in Benin was greater than or equal to 10 years.

Perhaps, the high illiteracy in this sector also portrays the status of education in the country. For instance, in Morocco and Ghana, where the literacy level is high with citrus production far higher than ours, Lahlali et al. [25] and Boakye et al. [26] reported that 17 % and 26 % respectively of citrus farmers are not literate. This shows that education is a determinant factor of agricultural productivity as it allows farmers to easily unpackage information related to farming technologies and apply them. Hence, efforts to provide agricultural information to citrus farmers should take into account the high illiteracy rate in this sector in Benin to develop materials in the local languages for technologies diffusion while promoting actions to improve the literacy rate.

Our results showed a wide variation in orchard density between and within the AEZs, ranging from 113.37 to 247.32 plants/ha. This could be explained by the low educational level and lack of technical support of the farmers. These results are similar to those of Gnimadi [27], who observed a trend from 160 to 360 plants/ha. AEZ VI recorded the highest average density. These results are in line with those of Akohoue et al. [28], who also recorded high densities of 260–271 plants/ha in two localities of the same AEZ. However, the high planting density combined with the absence of pruning practices hinders tree development, increases competition for sunlight and leads to low yields with small-sized fruit [29]. According to Afloukou et al. [30], the appropriate density range for citrus orchards in hot, humid regions like Benin is between 156 and 204 plants/ha. Bénaouf [31], pointed out in his report on organic citrus production that in tropical Africa, the normal orchard density is 202 plants/ha. This density, which is lower than that practiced by farmers, is linked to non-compliance with popularized densities. According to Kadir et al. [32], high crop densities contribute to the development and severity of phytopathogenic diseases. In orchards, they generate high humidity and less light under the canopy, which are favorable conditions for fungal or bacterial citrus disease development. Insect pest pressure is also high in crops at high densities.

The assessment of farmers' perceptions and knowledge showed that insect pests and diseases are the main constraints to citrus production in Benin. However, they have difficulty in identifying and differentiating insect and disease symptoms. In most cases, farmers acknowledge having observed the symptoms and damage caused by citrus fungal diseases in their orchards after seeing symptom photographs. These results confirm those of Lokossou et al. [10], who identified insect pests and diseases as major constraints to citrus orchard production in Benin, and expressed the need to build farmers’ capacity for early detection and disease management.

Using the pest guide, farmers were able to recognize the symptoms of anthracnose, black spot, brown rot, sooty mold and fruit rot that they encountered in their orchards. According to the farmers, these diseases had been observed for almost 20 years, but had become more remarkable in the last 5–6 years. Some 60 % of farmers confirmed the presence of black spot in their orchards, regardless of species. This disease causes enormous damage, attracting more attention from farmers than other diseases (anthracnose, brown rot, sooty mold and fruit rot), which are rarely observed in orchards. Although these fungal diseases are observed in orchards, farmers do not have a perfect understanding of when they appear. Nevertheless, 8–60 % of farmers, depending on the agro-ecological zone and the citrus species, report that these diseases are visible from the fruit enlargement stage until the fruit ripens (January to February and July to August), with major attacks in June, July, August and December. Low levels of attack were observed in May and November, according to the farmers. These results are consistent with those of Baldassari et al. [33], Lanza et al. [34] and Fialho et al. [14], who indicate that the first symptoms on fruit appear after fruit set until the ripening stages. The period of March, April and September mentioned by farmers corresponds to the months of flowering and fruit set for the major and minor crop [35].

Chemical control is the most common management method used by farmers. Several studies have reported that chemical control is the priority control method used by commercial farmers [36,37]. Various chemical products are used by citrus farmers in the areas studied, namely Acarius 018 EC (Abamectin 18 g/L), DD Force (Dichlorvos 1000 g/L EC), Lambda Super 2.5 EC (Lambda-Cyhalothrin 25 g/L), Pacha 25 EC (Lambda-Cyhalothrin 15 g/L + Acetamiprid 10 g/L), Pyro FTE 672 EC (Cypermethrin 72 g/L + Chlorpyrifos-Ethyl 600 g/L). Among these products, Pacha 25 EC is the most widely used by farmers. Pacha 25 EC is a chemical insecticide registered in Benin to manage defoliating and sucking pests of several crops such as cowpeas, cotton and vegetable crops. All the products cited by farmers to manage diseases are only insecticides, which they applied instead of fungicides. This justifies the farmers' statements that the chemical products applied are ineffective against the citrus fungal diseases reported. These results show that farmers do not differentiate between pesticide categories. The dominance of insecticides among the pesticides cited by farmers is indicative of the non-availability of fungicides and of farmers’ poor knowledge of citrus diseases. To effectively manage citrus fungal diseases, capacity-building for farmers in citrus disease recognition and management is needed. Citrus-specific pesticide registration is also necessary.

The majority of farmers indicated that climatic variability also affects the citrus sector, contributing to lower production and increased disease development. These results could be explained by the rarity and staggering of rainfall, the variation and high level of temperature and wind speed seen in the country today. The colonization of previously disease-free areas could also explain the development of these diseases and the losses recorded. However, the diseases could spread due to water stress and rising temperatures, which weaken the plant and predispose it to infection [12]. This could lead to yield losses and a decline in fruit quality, creating marketing and export constraints.

## 5 Conclusions

Several fungal diseases are associated with citrus in production zones (AEZ, V, VI, VII and VIII) in Benin. Among these diseases, black spot is perceived as the most damaging, causing greater yield losses under favorable conditions, coupled with an almost total absence of appropriate control methods. In addition, farmers need knowledge of good agronomic management and phytosanitary practices, in relation with climatic constraints prevailing in production zones. Workshops or training sessions specifically focused on citrus black spot are necessary for growers to better understand and manage the disease. These sessions will cover topics such as disease identification, symptoms, the life cycle of the pathogen and effective management strategies, which will be developed in the form of pamphlets, brochures, posters and videos. To ensure the success of this sector, capacity building for farmers is necessary, while taking into account the socio-economic impact of diseases.

## Data availability statement

The datasets used in this study are available from the corresponding author upon reasonable request.

## CRediT authorship contribution statement

**Goudjo Habib Toessi:** Writing – original draft, Methodology, Investigation, Formal analysis, Data curation, Conceptualization. **Rachidatou Sikirou:** Supervision, Conceptualization. **Elisée Georges Dadé Ler-N'ogn Amari:** Supervision, Conceptualization. **Esaïe Gandonou:** Software, Methodology, Formal analysis. **Afio Zannou:** Software, Methodology, Formal analysis. **Daouda Koné:** Supervision.

## Declaration of competing interest

The authors declare that they have no known competing financial interests or personal relationships that could have appeared to influence the work reported in this paper.
