# Supplementary material for: Farmers’ perceptions and management of citrus fungal diseases in Benin
Source: Heliyon. 2024 Jun 13;10(12):e32775. doi: 10.1016/j.heliyon.2024.e32775 (PMC11237933; doi:10.1016/j.heliyon.2024.e32775)
Supplement: Multimedia component 1 [file mmc1.docx]

**Farmers’ perceptions and management of citrus fungal diseases in Benin**

Individual questionnaire sent to citrus growers

Name of investigator (1= Eyitayo OGOUBI ; 2= Nathanaël AHOGA ; 3= Plotin AGONVONON ; 4=

Angelo DJEHOUNKE ; Habib TOESSI) _________________________________________________

Fiche N°/______/ Survey date /_____/______/______/

**1. General**

| Features | Terms and conditions | Enter the answer |
| --- | --- | --- |
| Department (**DEPART**) | 1= Atlantique, 2= Zou, 3= Couffo, 4= Mono, 5= Plateau | /_____/ |
| Communes (**COMM**) | 1= Kétou, 2= Djidja, 3= Aplahoué, 4= Allada, 5= Tori, 6= Zè, 7= Toviklin, 8= Dogbo, 9= Klouékanmey, 10= Covè, 11= Zakpota, 12= Zagnanando, 13= Toffo, 14= Lalo, 15= Zogbodomey, 16= Athiémé, 17= Bopa, 18= Lokossa, 19= Agbangnizoun | /_____/ |
| Village (**VILL**) | Write the answer in the following column | /_____/ |

**2. Socio-demographic characteristics of the respondent**

| Headings | | Code | Answer |
| --- | --- | --- | --- |
| Name of farmer (**NAM**) | | Write the answer in the following column | /_____/ |
| First name of farmer (**FIRSTNAM**) | | Write the answer in the following column | /_____/ |
| Contact (**NUM**) | | Write the answer in the following column | /_____/ |
| Age (**AGE**) | | Write the answer in the following column | /_____/ |
| Sex (**SEX**) | | 1=Male, 0=Female | /_____/ |
| Level of  education  (**INSTRU**) | Formal  education  (**EDUF**) | 0= None; 1= Primary; 2= Secondary; 3= University | /_____/ |
|  | Literate  (ALPHA) | 1=Yes, 0=No | /_____/  /_____/ |
|  |  | If literate, what can you do (**ALPHAFAIT**)? 1=read, 2=write,  3=both |  |
| Marital status (**STAMA**) | | 1=Married, 2=Divorced, 3=Widowed, 4=Single, 5=Other | /_____/ |
| Size of producer's household  (**TAMEPRO**) | | Write the answer in the following column | /_____/ |
| Respondent's position in the  household (**POSIT**) | | 1=Head of household, 2=Spouse, 3=Son/Daughter,  4=Relatives | /_____/ |
| Ethnicity (local language)  (**ETHN**) | | 1= Adja ; 2= Fon ; 3= Mina ; 4= Sahouè ; 5= Yoruba ; 6= Nago ; 7= Tori ; 8= Kotafon ; 9= Aïzo ; 10= Others to be specified. | /_____/ |
| Origin of respondent (**ORES**) | | 1= Aboriginal; 2= Allochthonous | /_____/ |
| Member of an agricultural  association (**ASSAG**) | | 1=Yes, 0=No | /_____/ |
| If yes, which one(s)  (**NASSAG**)? | | Write the answer in the following column |  |
| Member of a citrus producer  group (**GROUPA**) | | 1=Yes, 0=No | /_____/ |
| f so, which one  (**NGROUPA**)? | | Write the answer in the following column |  |
| Contact with extension  (**VULGAR**) | | 1=Yes, 0=No | /_____/ |
| If yes, which structure  (**NVULGAR**) | | 1= INRAB ; 2= ATDA ; 3= PADMA ; 4= IITA ; 5= ONG; 6= others to be specified | /_____/ |
| Main activity (**ACTIP**) | | 1= Agriculture ; 2= Livestock ; 3= Processing ; 5= Fishing ;  6= Trade ; 7= Craft ; 8= Employees ; 9= Other to be specified | /_____/ |
| Secondary activity (**ACTIS**) | | 1= Agriculture ; 2= Livestock ; 3= Processing ; 5= Fishing ;  6= Trade ; 7= Craft ; 8= Employees ; 9= Other to be specified | /_____/ |
| Years of experience in citrus  fruit production (**EXPAG**) | | Write the answer in the following column | /_____/ |
| **3. Characteristics of citrus orchards** | | | |
| Citrus varieties grown  (VARBAG)  1= yes; 0= no | | Pineapple | /_____/ |
|  |  | Valencia | /_____/ |
|  |  | Tangerine | /_____/ |
|  |  | Tangelo | /_____/ |
|  |  | Grapefruit | /_____/ |
|  |  | Lemons | /_____/ |
|  |  | Others to be specified | /_____/ |
| How old is your citrus grove  (**AGEP**)? | | Pineapple | /_____/ |
|  |  | Valencia | /_____/ |
|  |  | Tangerine | /_____/ |
|  |  | Tangelo | /_____/ |
|  |  | Grapefruit | /_____/ |
|  |  | Lemons | /_____/ |
|  |  | Others to be specified | /_____/ |
| How many citrus plantation  plots do you have (**PAPLAN**)? | | Enter quantity | /_____/ |
| How many parcel units  correspond to one hectare in  your locality (**EQUIV**) | | Write the answer in the following column | /_____/ |
| What is the surface area of  your citrus groves in plot  units? (**SUPAG**) | | Pineapple | /_____/ |
|  |  | Valencia | /_____/ |
|  |  | Tangerine | /_____/ |
|  |  | Tangelo | /_____/ |
|  |  | Grapefruit | /_____/ |
|  |  | Lemons | /_____/ |
|  |  | Others to be specified | /_____/ |
| How many citrus plants do you  have on a plot unit (**PAGUP**)? | | Write the answer in the following column | /_____/ |
| Specify the distance between  plants (**ECART**) | | Write the answer in the following column | /_____/ |
| Mode of access to land on  which citrus fruits are grown  (**MODAC**) | | 1=Shared inheritance, 2=Unshared inheritance, 3=Purchase,  4=Gift, 5=Lease, 6=Other | /_____/  /_____/  /_____/ |
| **4. Disease outbreaks and control methods**   \| What problems are you  experiencing in your citrus  groves? (**PROBLMAGRU**) \| \| 1= Diseases; 2= Insects \| \| \| \| \| \| \| \| \| /_____/ \| \| \| --- \| --- \| --- \| --- \| --- \| --- \| --- \| --- \| --- \| --- \| --- \| --- \| --- \| \| What diseases are you  encountering in your orchards  (**MAREV**) \| \| Pineapple \| Black spot  (*Phyllosticta*  sp.) (**Tn**) \| \| \| /_____/ \| \| \| \| \| \| \| \| Anthracnose: More  or less circular  brown to black spots  measuring  approximately 1.5  mm (*Colletotrichum* sp.) (**Co**) \| \| \| /_____/ \| \| \| \| \| \| \| \| Curvulariosis:  Brown spots all over  the surface of the  fruit and leaves  (*Curvularia* sp.) (**Cu**) \| \| \| /_____/ \| \| \| \| \| \| \| \| Fruit rot  (*Fusarium* sp.)  (**Fu**) \| \| \| /_____/ \| \| \| \| \| \| \| \| Other to be  specified (**Ap**) \| \| \| /_____/ \| \| \| \| \| \| \| \| Valencia \| Black spot  (*Phyllosticta*  sp.) (**Tn**) \| \| \| /_____/ \| \| \| \| \| \| \| \| Anthracnose: More  or less circular  brown to black spots  measuring  approximately 1.5  mm (*Colletotrichum* sp.) (**Co**) \| \| \| /_____/ \| \| \| \| \| \| \| \| Curvulariosis:  Brown spots all over  the surface of the  fruit and leaves  (*Curvularia* sp.) (**Cu**) \| \| \| /_____/ \| \| \| \| \| \| \| \| Fruit rot  (*Fusarium* sp.)  (**Fu**) \| \| \| /_____/ \| \| \| \| \| \| \| \| Other to be  specified (**Ap**) \| \| \| /_____/ \| \| \| \| \| \| \| \| Tangerine \| Black spot  (*Phyllosticta*  sp.) (**Tn**) \| \| \| /_____/ \| \| \| \| \| \| \| \| Anthracnose: More  or less circular  brown to black spots  measuring  approximately 1.5  mm (*Colletotrichum* sp.) (**Co**) \| \| \| /_____/ \| \| \| \| \| \| \| \| Curvulariosis:  Brown spots all over  the surface of the  fruit and leaves  (*Curvularia* sp.) (**Cu**) \| \| \| /_____/ \| \| \| \| \| \| \| \| Fruit rot  (*Fusarium* sp.)  (**Fu**) \| \| \| /_____/ \| \| \| \| \| \| \| \| Other to be  specified (**Ap**) \| \| \| /_____/ \| \| \| \| \| \| \| \| Tangelo \| Black spot  (*Phyllosticta*  sp.) (**Tn**) \| \| \| /_____/ \| \| \| \| \| \| \| \| Anthracnose: More  or less circular  brown to black spots  measuring  approximately 1.5  mm (*Colletotrichum* sp.) (**Co**) \| \| \| /_____/ \| \| \| \| \| \| \| \| Curvulariosis:  Brown spots all over  the surface of the  fruit and leaves  (*Curvularia* sp.) (**Cu**) \| \| \| /_____/ \| \| \| \| \| \| \| \| Fruit rot  (*Fusarium* sp.)  (**Fu**) \| \| \| /_____/ \| \| \| \| \| \| \| \| Other to be  specified (**Ap**) \| \| \| /_____/ \| \| \| \| \| \| \| \| Grapefruit \| Black spot  (*Phyllosticta*  sp.) (**Tn**) \| \| \| /_____/ \| \| \| \| \| \| \| \| Anthracnose: More  or less circular  brown to black spots  measuring  approximately 1.5  mm (*Colletotrichum* sp.) (**Co**) \| \| \| /_____/ \| \| \| \| \| \| \| \| Curvulariosis:  Brown spots all over  the surface of the  fruit and leaves  (*Curvularia* sp.) (**Cu**) \| \| \| /_____/ \| \| \| \| \| \| \| \| Fruit rot  (*Fusarium* sp.)  (**Fu**) \| \| \| /_____/ \| \| \| \| \| \| \| \| Other to be  specified (**Ap**) \| \| \| /_____/ \| \| \| \| \| \| \| \| Lemons \| Black spot  (*Phyllosticta*  sp.) (**Tn**) \| \| \| /_____/ \| \| \| \| \| \| \| \| Anthracnose: More  or less circular  brown to black spots  measuring  approximately 1.5  mm (*Colletotrichum* sp.) (**Co**) \| \| \| /_____/ \| \| \| \| \| \| \| \| Curvulariosis:  Brown spots all over  the surface of the  fruit and leaves  (*Curvularia* sp.) (**Cu**) \| \| \| /_____/ \| \| \| \| \| \| \| \| Fruit rot  (*Fusarium* sp.)  (**Fu**) \| \| \| /_____/ \| \| \| \| \| \| \| \| Other to be  specified (**Ap**) \| \| \| /_____/ \| \| \| \| \| \| \| \| Other to be  specified \| Black spot  (*Phyllosticta*  sp.) (**Tn**) \| \| \| /_____/ \| \| \| \| \| \| \| \| Anthracnose: More  or less circular  brown to black spots  measuring  approximately 1.5  mm (*Colletotrichum* sp.) (**Co**) \| \| \| /_____/ \| \| \| \| \| \| \| \| Curvulariosis:  Brown spots all over  the surface of the  fruit and leaves  (*Curvularia* sp.) (**Cu**) \| \| \| /_____/ \| \| \| \| \| \| \| \| Fruit rot  (*Fusarium* sp.)  (**Fu**) \| \| \| /_____/ \| \| \| \| \| \| \| \| Other to be  specified (**Ap**) \| \| \| /_____/ \| \| \| \| \| \| \| \| How many years have you been  observing these diseases in your  citrus grove? (**ANMAL**) \| \| Pineapple \| Tn /_____/ Co /_____/ Cu /_____/    Fu /_____/ Ap /_____/ \| \| \| \| \| \| \| \| \| \| \| Valencia \| Tn /_____/ Co /_____/ Cu /_____/  Fu /_____/ Ap /_____/ \| \| \| \| \| \| \| \| \| \| \| Tangerine \| Tn /_____/ Co /_____/ Cu /_____/  Fu /_____/ Ap /_____/ \| \| \| \| \| \| \| \| \| \| \| Tangelo \| Tn /_____/ Co /_____/ Cu /_____/    Fu /_____/ Ap /_____/ \| \| \| \| \| \| \| \| \| \| \| Grapefruit \| Tn /_____/ Co /_____/ Cu /_____/  Fu /_____/ Ap /_____/ \| \| \| \| \| \| \| \| \| \| \| Lemons \| Tn /_____/ Co /_____/ Cu /_____/  Fu /_____/ Ap /_____/ \| \| \| \| \| \| \| \| \| \| \| Other to be  specified \| Tn /_____/ Co /_____/ Cu /_____/    Fu /_____/ Ap /_____/ \| \| \| \| \| \| \| \| \| \| \| In which month of the year do  each of these diseases appear?  (**APAMAL**) \| \| Jan= 1 ; Feb= 2 ; Mar= 3 ; Avl= 4 ; Mai= 5 ; Jun= 6; Jul= 7; Aot= 8; Sep= 9; Oct= 10; Nov= 11; Dec= 12 \| Tn /_____/ Co /_____/ Cu /_____/  Fu /_____/ Ap /_____/ \| \| \| \| \| \| \| \| \| \| \| At what stage does each disease  appear (**STADMAL**)? \| \| 1= Flowering; 2=  Setting; 3= Fruit set;  4= Ripeness \| Tn /_____/ Co /_____/ Cu /_____/  Fu /_____/ Ap /_____/ \| \| \| \| \| \| \| \| \| \| \| What symptoms  have you observed  on the leaves,  flowers and fruit of  your citrus plants?  (**SYMPLAG**) \| 1= Black spots (*Phyllosticta* sp.): Small necrotic spots with a grey centre on leaves and fruit;  2= Anthracnose (*Colletotrichum* sp.): More or less circular brown to black spots measuring around 1.5 mm on leaves and fruit;  3= Curvulariosis (*Curvularia* sp.): Brown spots all over the surface of the fruit and leaves;  4= Fruit rot (*Fusarium* sp.).  5= Other to be specified \| \| \| Pineapple \| \| \| \| /_____/ \| \| \| \| \| \| Valencia \| \| \| \| /_____/ \| \| \| \| \| \| Tangerine \| \| \| \| /_____/ \| \| \| \| \| \| Tangelo \| \| \| \| /_____/ \| \| \| \| \| \| Grapefruit \| \| \| \| /_____/ \| \| \| \| \| \| Lemons \| \| \| \| /_____/ \| \| \| \| \| \| Other to be  specified \| \| \| \| /_____/ \| \| \| \| \| \| At what time of year are more  than 50% of your citrus plants  attacked? (**FORATAQ**) \| \| Jan= 1 ; Feb= 2 ; Mar= 3 ; Avl= 4 ; Mai= 5 ; Jun= 6; Jul= 7; Aot= 8; Sep= 9; Oct= 10; Nov= 11; Dec= 12 \| Pineapple \| \| /_____/ \| \| \| \| \| \| \| \| \| Valencia \| \| /_____/ \| \| \| \| \| \| \| \| \| Tangerine \| \| /_____/ \| \| \| \| \| \| \| \| \| Tangelo \| \| /_____/ \| \| \| \| \| \| \| \| \| Grapefruit \| \| /_____/ \| \| \| \| \| \| \| \| \| Lemons \| \| /_____/ \| \| \| \| \| \| \| \| \| Other to be  specified \| \| /_____/ \| \| \| \| \| \| \| \| \| At what time of year are less  than 50% of your citrus plants  attacked? (**FAIBATAQ**) \| \| Jan= 1 ; Feb= 2 ; Mar= 3 ; Avl= 4 ; Mai= 5 ; Jun= 6; Jul= 7; Aot= 8; Sep= 9; Oct= 10; Nov= 11; Dec= 12 \| Pineapple \| \| /_____/ \| \| \| \| \| \| \| \| \| Valencia \| \| /_____/ \| \| \| \| \| \| \| \| \| Tangerine \| \| /_____/ \| \| \| \| \| \| \| \| \| Tangelo \| \| /_____/ \| \| \| \| \| \| \| \| \| Grapefruit \| \| /_____/ \| \| \| \| \| \| \| \| \| Lemons \| \| /_____/ \| \| \| \| \| \| \| \| \| Other to be  specified \| \| /_____/ \| \| \| \| \| \| \| \| \| At what time of year are citrus  plants not attacked at all?  (**PASATAQ**) \| \| Jan= 1 ; Feb= 2 ; Mar= 3 ; Avl= 4 ; Mai= 5 ; Jun= 6; Jul= 7; Aot= 8; Sep= 9; Oct= 10; Nov= 11; Dec= 12 \| Pineapple \| \| /_____/ \| \| \| \| \| \| \| \| \| Valencia \| \| /_____/ \| \| \| \| \| \| \| \| \| Tangerine \| \| /_____/ \| \| \| \| \| \| \| \| \| Tangelo \| \| /_____/ \| \| \| \| \| \| \| \| \| Grapefruit \| \| /_____/ \| \| \| \| \| \| \| \| \| Lemons \| \| /_____/ \| \| \| \| \| \| \| \| \| Other to be  specified \| \| /_____/ \| \| \| \| \| \| \| \| \| What methods do you use to  combat the various diseases?  (**METHOLUT**) \| \| 1= Nothing; 2= Chemical; 3= Endogenous measure; 4= Other to be  specified \| \| \| /_____/ \| \| \| \| \| \| \| \| \| If 3 for code **METHOLUT**,  describe \| \|  \| \| \|  \| \| \| \| \| \| \| \| \| If 4 for code **METHOLUT**,  describe \| \|  \| \| \|  \| \| \| \| \| \| \| \| \| If 2 for the **METHOLUT** code,  what are the names of the  products used? (**NOMPRO**) \| \| 1)  2)  3) \| \| \| \| \| \| \| \| \| \| \| \| f 2 for code **METHOLUT**,  How many cans do you use  during the year (**BOUTAN**)? \| \| 1)  2)  3) \| \| \| \| \| \| \| \| \| \| \| \| If 2 for code **METHOLUT**,  What is the capacity of a box? \| \| 1)  2)  3) \| \| \| \| \| \| \| \| \| \| \| \| If 2 for code **METHOLUT**,  where do you buy the products  (**ACHPRO**) \| \| 1)  2)  3) \| \| \| \| \| \| \| \| \| \| \| \| If 2 for code **METHOLUT**,  What is the purchase price of the  products? (**PRIACHAP**) \| \| 1)  2)  3) \| \| \| \| \| \| \| \| \| \| \| \| If 2 for code **METHOLUT**,  How many treatments do you  have during the year  (**TRAITAN**) \| \|  \| \| \| \| \| \| \| \| \| \| \| \| If 2 for code **METHOLUT**,  What is the surface area treated  (**SUPTRA**) \| \|  \| \| \| \| \| \| \| \| \| \| \| \| If 2 for code **METHOLUT**, How long have you been  treating your orchards  (**QUATRAVER**) \| \|  \| \| \| \| \| \| \| \| \| \| \| \| If 2 for code **METHOLUT**, What parts are covered? **(PARTRAIT)** \| \| 1= Leaf; 2= Fruit; 3= Stem; 4= Soil; 5= Whole plant \| \| \| \| \| \| \| /_____/ \| \| \| \| \| If 2 for code METHOLUT,  Do you use preventive treatment  (**PREVENTRAIT**)? \| \| 1= Yes ; **0=** No \| \| \| \| \| \| \| /_____/ \| \| \| \| \| If 2 for the METHOLUT code,  what is your source of information on the products?  (**SOURCE**) \| \| 1= INRAB ; 2= ATDA ; 3= PADMA ; 4= IITA ; 5= ONG ; 6= Other farmer \| \| \|  \| \| \| \| \| \| \| \| \| If 2 for the METHOLUT code,  what is the year of product  information (**ANINF**)? \| \| Inscrire la réponse dans la colonne suivante \| \| \| \| \| \| \| \| /_____/ \| \| \| \| If 2 or 3 for code METHOLUT, have you followed one or more training courses on citrus disease management? (**FORMATION**) \| \| 1=Yes, **0=**No \| \| \| \| \| \| \| \| \| \| /_____/ \| \| If so, what was it about? (**INTITUL**) \| \| Write the answer in the following column \| \| \| \| \| \| \| \| \| /_____/ \| \| \| If 2, 3 or 4 for the **METHOLUT** code, how effective are the control measures? (**EFFECTIVE**) \| \| 1= very effective, 2= moderately effective, 3=  not very effective, 4= not effective, 5= other to  be specified \| \| \| \| \| /_____/ \| \| \| \| \| \| \| Do you use labour ? (**MAINOEV**) \| \| 1= Yes ; **0=** No \| \| \| \| \| /_____/ \| \| \| \| \| \| \| If so, how long have you been using labour? (**QUAMAINOEV**) \| \| Write the answer in the following column \| \| \| \| \| /_____/ \| \| \| \| \| \| \| If so, what is the cost of labour over the year ?  (**COUMAINOEV**) \| \| Write the answer in the following column \| \| \| \| \| /_____/ \| \| \| \| \| \| \| Based on your observations over the last 10 years, has citrus production decreased or increased in your locality?  (**PRODIMAUG**) \| \| 1= Increased  2= Decreased  3= No change  4= No idea \| Pineapple \| \| /_____/ \| \| \| \| \| \| \| \| \| Valencia \| \| /_____/ \| \| \| \| \| \| \| \| \| Tangerine \| \| /_____/ \| \| \| \| \| \| \| \| \| Tangelo \| \| /_____/ \| \| \| \| \| \| \| \| \| Grapefruit \| \| /_____/ \| \| \| \| \| \| \| \| \| Lemons \| \| /_____/ \| \| \| \| \| \| \| \| \| Other to be  specified \| \| /_____/ \| \| \| \| \| \| \| \| | | | |
| **6. Farmers' assessment of climate variability and the citrus cycle**   \| In your experience, are the seasons getting wetter or drier (**SEASON**)? \| 1= Rainy 2= Dry \| \| /_____/ \| \| \| --- \| --- \| --- \| --- \| --- \| \| What is the intensity of the rain? (**INTENSITE**) \| 1= Increasingly strong; 2=Increasingly weak \| \| /_____/ \| \| \| Can you tell us about the times when you notice more symptoms of fungal diseases on citrus fruit and/or leaves? **(DMSYMPLUS)** \| Temperature \| 1= High; 2= Low \| \| /_____/ \| \| Humidity \| 1= High; 2= Low \| \| /_____/ \| \| Wind \| 1= Very violent; 2=Violent; 3= Not violent \| \| /_____/ \| \| Can you tell us about the times when you don't notice any symptoms of fungal diseases on citrus fruit and/or leaves? **(DMPASYM)** \| Temperature \| 1= High; 2= Low \| \| /_____/ \| \| Humidity \| 1= High; 2= Low \| \| /_____/ \| \| Wind \| 1= Very violent; 2=Violent; 3= Not violent \| \| /_____/ \| \| Has the length of the citrus cycle changed? **(LCYCLE)** \| 1= Yes ; 0= No \| \| /_____/ \| \| \| If yes, indicate (in months) (**PLCYCLE**) \| Length of previous cycle \| \|  \| \| \| Current cycle length \| \|  \| \| \| Has the flowering date also changed (**FLORA**) \| 1= Yes ; 0= No \| \| /_____/ \| \| \| If yes, indicate (in months) (**PFLORA**) \| Earlier date \| \|  \| \| \| The current date \| \|  \| \| \| Has the date of physiological maturity changed? **(MATUR)** \| 1= Yes ; 0= No \| \| /_____/ \| \| \| If yes, indicate (in months) (**PMATUR**) \| Earlier date \| \|  \| \| \| The current date \| \|  \| \| \| What is the effect of climate change on citrus fruit production (**CCPROD**) \| 1= Increase in production ; 2= Decrease in production ; 3= Constant production ; 4= Other to be specified \| \| /_____/ \| \| \| What is the effect of climate change on the development of diseases (**CCMAL**) \| 1= Increase in symptoms; 2= Decrease in symptoms; 3= Constant level of symptoms; 4= Other to be specified \| \| /_____/ \| \| | | | |

**Geographical coordinates (COGEO)**
